# Parent-Teen Communication about Sexual and Reproductive Health: Cohort Differences by Race/Ethnicity and Nativity

**DOI:** 10.3390/ijerph16050833

**Published:** 2019-03-07

**Authors:** Hannah Lantos, Jennifer Manlove, Elizabeth Wildsmith, Bianca Faccio, Lina Guzman, Kristin A. Moore

**Affiliations:** 1 Youth Development Program Area, Child Trends, 7315 Wisconsin Ave, Suite 1200W, Bethesda, MD 20814, USA; kmoore@childtrends.org; 2 Reproductive Health and Family Formation Program Area, Child Trends, 7315 Wisconsin Ave, Suite 1200W, Bethesda, MD 20814, USA; jmanlove@childtrends.org (J.M.); ewildsmith@childtrends.org (E.W.); bfaccio@childtrends.org (B.F.); lguzman@childtrends.org (L.G.)

**Keywords:** sexual and reproductive health, teenagers, Hispanics, parenting, condom use, contraception, sex education

## Abstract

Parent-teen discussions about sexual and reproductive health (SRH) are associated with delayed sex and higher contraceptive use among teens. Using the National Survey of Family Growth, we conducted bivariate and multivariate analyses of different types of parent-teen SRH discussions among two cohorts of teens. We describe differences in patterns for males and females by race/ethnicity and nativity, and test for racial/ethnic interactions within each cohort. Analyses found that the prevalence of parent-teen discussions about SRH increased across cohorts. For males and females, there were increases in parent-teen discussions about condoms, and for males only, there were increases in any SRH discussions and discussions about contraception and STIs. Based on interactions, parent-teen discussions and STI discussions increased most for Hispanic females, and among Hispanics, increased most for the foreign-born. These data indicate increases in different types of parent-teen SRH discussions, particularly for males and foreign-born teens overall, and for Hispanic teen females regarding condom use. Future research should examine what factors are driving these changes, including changes in the structure of U.S. Hispanic communities and expansion of evidence-based teen pregnancy prevention programs.

## 1. Introduction

Adolescence is a critical developmental period when youth begin to develop their romantic and sexual identities and is an important time to learn about how to engage in healthy romantic and sexual behavior, which then sets the stage for healthy adult relationships. Additionally, adolescence is a time to focus health promotion efforts on reducing the risk of negative sexual health outcomes, such as teen births and sexually transmitted infections (STIs). Although teen pregnancy and birth rates have declined across all racial and ethnic groups in the U.S. since the early 1990s [1,2], rates among black and Hispanic teens are still more than twice as high as rates among white teens. Additionally, STIs have increased in recent years and large racial and ethnic disparities in STIs, also persist [3,4]. Discussions about healthy romantic and sexual relationships are part of a comprehensive approach to sex education supported by physicians [5,6] and public health professionals [7].

Parent-teen discussions may be particularly important, especially when it comes to reducing engagement in sexual risk behaviors. Researchers have found that when teens—particularly girls—talk to their parents about sexual behaviors, contraception, STIs, and pregnancy prevention (from here on, SRH discussions), they are more likely to engage in safe sexual behaviors, including abstinence and protective behaviors that prevent pregnancy and STIs [8]. For example, teens who have high levels of communication with their parents are more likely to delay first sexual intercourse [9], discuss pregnancy and STI prevention with sexual partners [10], use contraception [9], and use condoms at first and most recent intercourse [11]. While mothers tend to be the primary communicators with teenagers about sexual behaviors [11,12,13], father-teen communication is also linked to reduced risky behaviors [14]. Importantly, teens themselves believe in this association [15]. A recent poll found that nearly nine out of ten teens believe that open and honest conversations with parents about sex can help them avoid a pregnancy [16].

There are differences in the frequency and content of parent-teen discussions about SRH by race and Hispanic ethnicity, which may help explain some of the disparities in sexual health outcomes. Some research has found that Hispanic mothers report engaging in fewer discussions about sexual risk behaviors with their teens than white and black mothers [17,18], while other research has found that both black and Hispanic teens spoke to their parents less often about sex than white teens [19]. Interestingly, Hispanic mothers report feeling less comfortable talking about sex with their children than mothers in other races or ethnic groups, but are also more likely to correctly report whether their children are sexually active, suggesting that they may, in fact, be communicating with their children about their sexual behaviors but perhaps in less obvious or intentional ways [17]. 

To improve reproductive health outcomes, recent federal initiatives have acknowledged the importance of, and encouraged, parent-teen conversations about SRH. Healthy People 2020, which sets national health priorities, includes a family planning goal to increase the number of teens who talk to their parents about abstinence, birth control methods, HIV, and STIs [20]. Additionally, the federal government has included recommendations for increasing parent-teen dialogue in many recent pregnancy prevention programs [21,22,23]. The evidence-based Teen Pregnancy Prevention (TPP) program and the Personal Responsibility and Education Program (PREP) are federal initiatives that have funded organizations across the country since 2010 to reduce teen pregnancy [24,25]. As an example of efforts to support discussions between parents and teens, Families Talking Together, an intervention that works to improve parent-child communication and monitoring, was recently added to the approved federal list of evidence-based teen pregnancy prevention programs [26]. Communities with high teen birth rates, which are often low-income and/or communities of color, are the focus of many of these pregnancy prevention efforts [21,22,23]. 

It is not yet known whether these recent federal efforts have resulted in increased parent-teen discussions about SRH and, as a result, have helped reduce sexual risk behaviors and racial/ethnic disparities. In this paper, we build on prior research [27] and examine the changing prevalence of parent-teen discussions about SRH across two recent cohorts using data from the National Survey of Family Growth (NSFG). Using multivariate analyses, we: (1) focus on recent cohorts of youth (through 2015), (2) look separately at patterns by race and Hispanic ethnicity, and (3) examine multiple types of parent-teen discussions (including discussions about how to say no to sex, contraception, sexually transmitted infections or STIs, and condom use). The Hispanic population is very diverse, and well-established differences in health and sexual risk behaviors exist by nativity [28,29]—comparing teens who were born inside or outside the U.S. Because of this, we also test for nativity differences within the Hispanic population (the sample sizes for white and black foreign-born respondents were low, so we did not conduct separate analyses by nativity status for these populations). We also stratify analyses by gender because, although both sons and daughters both can benefit from conversations with their parents [27,30,31], boys report fewer discussions than girls [8]. Additionally, research finds that the content of sexual health conversations with parents differs by gender (of both the parent and the child) [8,32]. For example, parents are more likely to highlight the negative repercussions of sexual activity for daughters, while they tend to focus on preventing pregnancy or disease for sons [8].

## 2. Materials and Methods

The NSFG is a continuous, nationally representative survey of men and women of reproductive age in the United States that collects information on fertility, contraceptive use, health, and more [33]. For this paper, we used the public use data files for male and female respondents in the 2006–2010 cohort (“Cohort 1”) and the 2011–2015 cohort (“Cohort 2”). We first limited our analytic sample to the 8796 teenagers ages 15 to 19 in Cohorts 1 (*n* = 4662) and 2 (*n* = 4134). We then removed 867 teens who were not categorized as white, black, or Hispanic as the sample sizes are small but also because this is a varied group with different degrees of social and economic disadvantage. This resulted in a final analytic sample of 7929 teens across Cohorts 1 (*n* = 4224) and 2 (*n* = 3705) and included 4019 females and 3910 males. Because of differences in missing data on outcomes, our sample sizes vary slightly across models. (We used *t*-tests to test for differences in the levels of covariates by missingness on the outcome variables and determined that there were no differences, so sub-population sizes in the regressions vary slightly based on respondents who were missing the specific outcome variable values.)

### 2.1. Measures

Our primary outcome of interest is parent-teen conversations about sexual and reproductive health. Respondents reported whether they had ever spoken with their parents or guardians before the age of 18 about six different topics: how to say no to sex, contraceptive methods, where to obtain contraception, STIs, how to prevent HIV, and how to use a condom. We used these data to develop five binary dependent variables (1 = yes). The first variable (Any SRH Topic) measures whether respondents discussed any of these SRH topics with a parent. The second (Saying no to sex) measures whether respondents ever spoke to a parent about how to say no to sex. The third (Contraception) measures whether respondents talked with a parent about methods of contraception and/or where to obtain contraception. The fourth (STIs) measures whether respondents discussed STDs or HIV with their parents. The fifth (Condoms) measures whether respondents discussed how to use a condom with a parent. (We considered grouping condoms with the STI variable because research suggests that condom conversations are often related to disease prevention and are often cursory. However, Vanderberg et al. (2016) grouped them with contraception. Therefore, we break them out into three variables—contraception, STIs/HIV, and condoms.

Our analyses aim to describe patterns of teen-parent communication for males and females across three key independent variables: (1) race/ethnicity, (2) cohort, and (3) Hispanic nativity. Race/ethnicity is a three-category variable based on respondents’ reports identifying themselves as non-Hispanic white (white), non-Hispanic black (black), or Hispanic. Cohort is a binary variable which indicates which survey the data came from—2006–2010 (0) or 2011–2015 (1). Nativity is used in the final model to differentiate between Hispanic teens born in the U.S. (0) or abroad (1). Gender indicates whether the respondent self-identified as male (0) or female (1).

There are a range of other individual and background factors that are likely linked to teen-parent conversations about sexual health, race/ethnicity, and cohort. To ensure that any observed patterns in our outcome measures are not being driven by variation in these factors, we include controls for: age; whether the respondent is currently enrolled in school or working (a measure of youth disconnection); mother’s education (less than high school vs. high school vs. some college or more); family structure at age 14 (living with two parents or not); the importance of religion (very important vs. somewhat/not important); and whether the respondent reported ever having had sex [32,34,35,36,37].

### 2.2. Analysis

First, we calculated the demographic characteristics of the sample and tested for gender and racial/ethnic differences (Table 1). Second, we calculated the unadjusted, weighted changes in parent-teen communication by race/ethnicity and gender across cohorts. We ran bivariate logistic regressions to assess significant differences across cohort and gender (Table 2).

Third, we calculated multivariate logistic regressions for the five outcomes. For each outcome, we estimated two regression models: one controlling for all covariates and a second with an interaction between cohort and race/ethnicity. Interaction analyses allowed us to test whether changes across cohorts in parent-teen communication varied by race/ethnicity. All of these analyses were run separately by gender (Table 3 and Table 4). Finally, we ran a pooled model with both genders to test for differences between U.S.-born and foreign-born Hispanics (Table 5). We pooled across gender because of the low sample size of foreign-born Hispanics. The final model includes an interaction between cohort and foreign-/U.S.-born status to test whether patterns observed over time for Hispanics differed by nativity. To ease interpretability, predicted probabilities were calculated for interactive models using the mean value of all covariates.

NSFG provided survey weights were used in all analyses. We conducted all analyses in Stata 13.1 and tested all variables in the models for multicollinearity using the collin command in Stata finding no evidence of inflated variance.

## 3. Results

Table 1 shows the weighted distributions of control variables by gender and by cohort, noting any significant cohort differences. Approximately 16 percent of teens in the sample were black, 20–26 percent were Hispanic, four in ten were ages 18–19, six in ten grew up in two-parent families, and more than half had mothers who had completed some college or more. These characteristics did not vary much across cohorts, although there were significant declines in the percentage of female teens who were disconnected and a significant increase in the percentage of boys whose mother had less than a high school degree.

Table 2 presents cohort and racial/ethnic differences in parent-teen discussions by gender. In both cohorts, approximately 8 in 10 female teens reported talking with a parent about any SRH topic. Among females, there were no significant changes in discussions about any of the SRH topics across cohorts with one exception; there was a 6.4 percentage point increase in discussions about how to use a condom (from 30.4 percent in 2006–2010 to 36.8 percent in 2011–2015). In contrast, for males, discussions about any SRH topic increased by 15 percentage points between the 2006–2010 and 2011–2015 cohorts and there were increases, though smaller, in talking about contraception (4.9 percentage points), STIs (6.4 percentage points), and condoms (7.8 percentage points).

Among females, there were a few racial/ethnic differences in patterns over time. There was a significant increase across cohorts in talking about condom use among Hispanic females, both U.S. and foreign-born. Additionally, foreign-born Hispanic females reported an increase in discussions about STIs. Among males, Hispanics saw significant increases in conversations about saying no and STIs, while white males saw significant increases in discussions about STIs and condoms.

There were also notable racial/ethnic differences in parent-teen discussions for both genders *within* each cohort (significance based on a *p*-value less than or equal to 0.05 is noted below the race/ethnicity rows in Table 2 with the letter “a”). Among females, there were significant differences across racial and ethnic groups in talking about each SRH topic. Across every topic, Hispanic females in Cohort 1 were least likely to talk to their parents, while black females were generally the most likely (except whites were most likely to discuss contraception). In Cohort 2, Hispanic girls fell in between black and white females any discussions as well as discussions about STIs and condoms.

Among males, there were racial/ethnic differences in seven of the ten outcomes across the two cohorts. In Cohort 1, there were differences in discussing any SRH topic, with black boys being the most likely to talk to their parents (78.2 percent) and white boys being the least likely (66.7 percent). For this cohort there were also differences in discussions about how to say no to sex and about STIs. Only 29.4 percent of Hispanic males in Cohort 1 talked to their parents about how to say no to sex, compared to 45.3percent of white males; meanwhile, 48.7 percent of white males talked about STIs compared to 65.7 percent of black teens. There were no racial/ethnic differences for males in either cohort in talking about contraception. In Cohort 2, Hispanic males were the least likely to report talking to their parents about how to say no to sex, and both Hispanic and black males reported more conversations about STIs than white males. 

For Hispanic males and females in Cohort 1, a higher percentage of U.S.-born Hispanics reported having any discussions with their parents than did foreign-born Hispanics. In addition, U.S.-born females were more likely than foreign-born females to discuss STIs with their parents and U.S.-born males were more likely to discuss how to say no to sex. 

Table 3 and Table 4 show results from the multivariate regression analyses examining factors associated with five types of parent-teen discussions for females (Table 3) and males (Table 4). Table 3 shows that females in Cohort 2 had higher odds of talking with parents about how to use a condom (OR = 1.4) than females in Cohort 1, net of controls. For conversations about STIs and condoms, black females had higher odds of talking to their parents than white females (OR = 1.6 and 1.7, respectively). In addition, both Hispanic and black teens had lower odds than white teens of talking to their parents about contraception.

Several of the covariates were also associated with parental discussions for females. Higher religiosity was associated with greater odds of discussing how to say no to sex (OR = 1.4) and lower odds of having a conversation about contraception or condoms (OR = 0.8 and 0.7, respectively) relative to lower religiosity. Disconnected teens (vs. those who were in school or working) had lower odds of talking to parents about any SRH topic as well as several of the specific SRH topics. Living with two biological or adoptive parents (vs. living with none or one parent) was associated with lower odds of discussing condom use (OR = 0.7), while being older was associated with lower odds of discussing HIV/STIs (OR = 0.8). Ever having had sex was associated with increased discussions in each category except for how to say no to sex. Some covariates were significantly associated with parent-teen discussions in bivariate analyses (not shown here), but lost significance in multivariate analyses, net of all controls. For example, higher maternal education was positively associated with discussions about any SRH topic in bivariate, but not multivariate, analyses, while growing up in a two-parent family was negatively associated with discussions about birth control and STIs in bivariate analyses only. In contrast, the measure of disconnected youth became negatively associated with discussing birth control and HIV in the multivariate analyses but was not significant in bivariate analyses.

**Figure 1:**
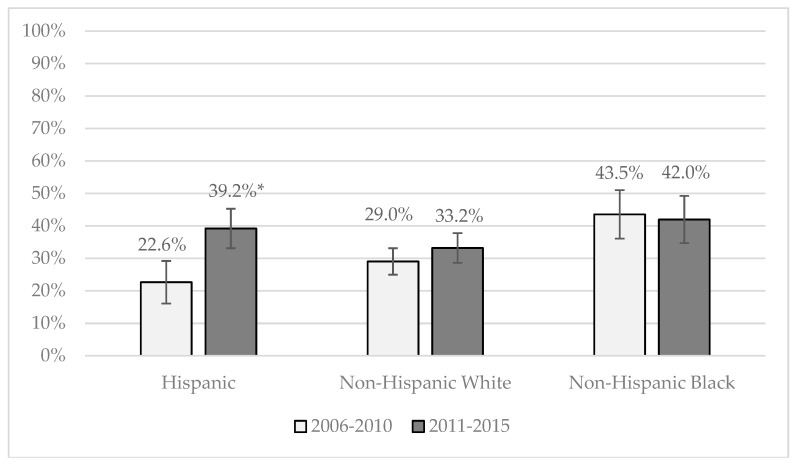
Predicted probability of parent-teen discussions about how to use a condom by cohort and race/ethnicity, among females. Significant differences between cohorts with *p*-values < 0.05 are marked with an *.

We also ran models that included an interaction term between race/ethnicity and cohort. These interaction terms were only significant for females in one case (predicted probability results are shown in Figure 1 below but are not shown in Table 2). Among females, the interaction between Cohort 2 and Hispanic ethnicity for discussions about condom use (OR = 1.8) were significant. This interaction indicates that there were especially high increases in the prevalence of discussions about condom use across cohorts for Hispanic females compared with white females. 

Figure 1 shows the racial/ethnic specific predicted probabilities of teens discussing how to use condoms with their parents across the two cohorts (predicted probabilities were assessed at the mean values for all controls.) As shown in Figure 1, our model predicts that in Cohort 1, 22.6 percent of Hispanic females spoke to their parents about using condoms. In Cohort 2, 39.2 percent of Hispanic females did. There were no significant cohort differences for white or black females making this 16.6 percentage point increase striking. 

Table 4 shows results from the multivariate analyses for males. Unlike females, we see sustained cohort differences net of controls. Specifically, males in Cohort 2 had greater odds of having discussions with their parents about any SRH topic (OR = 2.2), about STIs/HIV (OR = 1.2), and about condom use (OR = 1.3) compared with males in Cohort 1, net of controls. Black and Hispanic males had greater odds of talking to their parents about any SRH topic and of talking with their parents about STIs than white males. Hispanic males had 30 percent lower odds of talking to their parents about how to say no to sex than white males. Black males had higher odds of discussing how to use condoms (OR = 1.7) than white males. 

Like females, several control variables were linked to parent-teen discussions. Disconnected teens (who were not in school or working) had lower odds of talking to parents about how to say no to sex (OR = 0.6) and contraception (OR = 0.7), and higher religiosity was associated with higher odds of talking to parents about how to say no to sex (OR = 1.7). Older teens had greater odds of talking with their parents about contraception (OR = 1.3), but 20 percent lower odds of talking about STIs/HIV and condom use. Additionally, having a more educated mother was associated with all outcomes except condom use, and ever having had sex was associated with all outcomes except saying no to sex. There were no significant interactions between cohort and race/ethnicity for males. Like for females, some covariates were significantly associated with parent-teen discussions in bivariate analyses (not shown here), but lost significance in multivariate analyses, net of all controls. For example, family structure is significantly associated with talking about how to say no to sex in the bivariate model but not in the multivariate analyses.

Table 5 shows differences among Hispanic teens by nativity (males and females combined). There are statistically significant increases among Hispanics in talking to parents about any SRH topic, about STIs or HIV, and about condoms across cohorts. There were significant increases across cohort in any SRH discussions (OR = 1.7), STIs (OR = 1.3), and condom use (OR = 1.5). Females had higher odds of talking to their parents about how to say no to sex (OR = 2.7) and contraception (OR = 2.2), while they had lower odds of discussing how to use condoms (OR = 0.6). We do not see significant differences by nativity status in this table, but the figures and text below show results from interaction models. 

**Figure 2:**
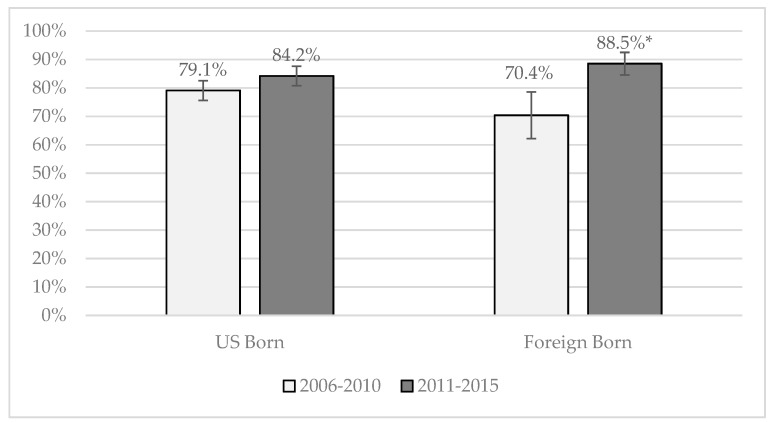
Predicted probability of parent-teen discussions about any SRH topic by nativity among male and female Hispanics. Significant differences between cohorts at the 5 percent level are marked with an *.

**Figure 3:**
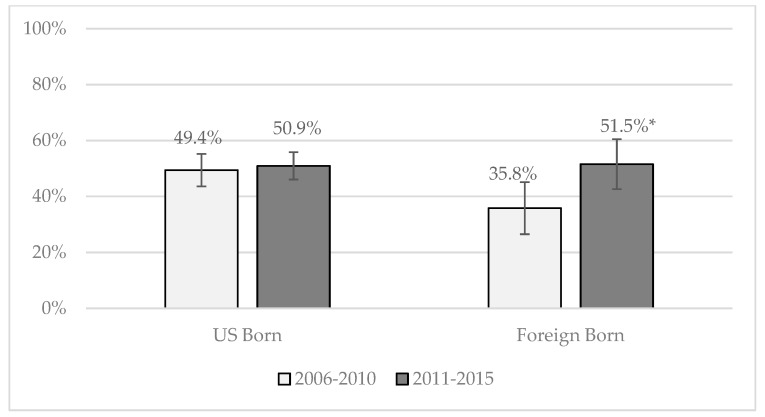
Predicted probability of parent-teen discussions about how to say no to sex by nativity among male and female Hispanics. Significant differences between cohorts at the 5 percent level are marked with an *.

Figure 2 and Figure 3 show the predicted probabilities, assessed at the mean values of controls, of the two models that had significant interaction terms (between nativity and outcome) for Hispanics across cohorts. Two models had significant interaction terms: talking to one’s parents about any SRH topic (OR = 2.3) and about how to say no to sex (OR = 1.8). As shown in Figure 2, 88.5 percent of Cohort 2 foreign-born Hispanics spoke to their parents about any SRH topic compared to 70.4 percent of foreign-born Hispanic teens in Cohort 1. This 18.1 percentage point increase was significantly greater than the 5.1 percentage point increase for U.S.-born Hispanic teens across cohorts. Similarly, for talking to parents about how to say no to sex, foreign-born Hispanic teens in Cohort 1 reported significantly fewer discussions than U.S.-born Hispanic teens (35.8 percent versus 49.4 percent, respectively in Figure 3). By Cohort 2, this difference had disappeared due to increases in discussions with parents among the foreign-born teens.

## 4. Discussion

Accurate and developmentally appropriate education on sexuality and healthy relationships can promote and support healthy sexual development and lay the groundwork for lifelong sexual health and wellbeing. Notably, research suggests that for teens, engaging in healthy conversations about SRH with their parents may help reduce the risk of engaging in sexual risk behaviors that can lead to unintended pregnancy and STIs [9,10]. This paper examined changes across cohorts in teens engaging in these conversations with their parents, while looking closely at differences by race/ethnicity as well as by nativity among Hispanic teens. We highlight several findings below.

First, we saw an overall increase in parent-teen discussions about any SRH topic, contraception, STIs, and condom use across the two cohorts for males, while only discussions about condom use increased for females. Notably, the vast majority of teens (78.2 percent of females and 84.5 percent of males) in the most recent cohort (2011–2015) reported having some type of conversation with their parents about SRH. Multivariate models confirmed cohort increases in parent-teen discussions on any SRH topic for males, STIs for males, and condoms for males and females net of all controls, indicating that these increases were not due to compositional differences across cohorts in any of our control measures. Interestingly, prior research on youth ages 15-17 documented declines in the prevalence of conversations about birth control and STIs between 1995 and 2002 [38,39].

Second, we found differences by race and ethnicity in teen-parent discussions about SRH topics. In Cohort 1, black and white females consistently reported more conversations with their parents about a number of sexual and reproductive health topics than Hispanic females. However, Hispanic females showed especially large increases in parent-teen discussions about condom use across cohorts. Interestingly, these increases in parent-teen discussions parallel dramatic declines in pregnancy as well as births among Hispanic teens over this same time period [40]. Though likely attributable to a number of factors—including changes in attitudes and economic factors—reduced birth rates may also be influenced by increased parent-teen communication about condoms. Future research could further explore the role that parental communication plays in these declines in teen births among Hispanics and other groups.

Separate analyses of Hispanics by nativity found especially large increases in reported parent-teen discussions about any SRH topic and how to say no to sex among foreign-born teens. It is unclear why we see this, though changes in immigration may be playing a role by changing the composition of foreign-born teens over time. Immigration to the U.S. has slowed dramatically over the past decade [41,42], meaning that more recent cohorts of foreign-born teens have been in the U.S. for a relatively longer period of time, on average, than those who were foreign-born in previous cohorts (high levels of missing data on this NSFG measure in our sample preclude its use). Research generally finds that teens who come to the U.S. at younger ages, or who have been in the U.S. for a longer period of time, tend to look more like their U.S. born counterparts than do those who come at older ages or more recently [43]. However, the short time period of these analyses suggests other factors could be playing a role. Future data will allow us to assess whether high rates of parent-teen discussions reported by foreign-born teens are sustained.

Third, while differences across race/ethnicity in the summative “any SRH topic” variable disappeared in the most recent cohort, racial/ethnic differences in conversations about specific topics persist, net of controls. The higher rates of discussions about STIs and condoms among parents of black and (sometimes) Hispanic teens may be because parents are aware of the higher rates of STIs in these populations and recognize the need to support their children [3,4,44]. In addition, Hispanic and black females continue to have lower odds (relative to white females) of discussing contraception with their parents. Previous quantitative research has highlighted more discomfort discussing sexual behaviors among Hispanic and black mothers than among white mothers [19], and qualitative research has found that Hispanic parents are more comfortable telling their teens to abstain from sex and avoid pregnancy than discussing how to use contraception and prevent pregnancy once they become sexually active [45]. Parents of color, many of whom report feeling more uncomfortable with discussing specific behaviors or talking about protection or pleasure [45], may have an easier time with conversations that are focused on avoiding negative outcomes than on the more positive aspects of sexual behavior. Exploring more of the content taking place in these conversations is an important next step for this research.

Finally, our analyses identified some key predictors of parent-teen discussions. Higher maternal education was consistently associated with greater likelihood of parent-teen discussions for males (and females in one case), suggesting that parent involvement efforts should focus particularly on populations with lower parental education. Consistent with prior research [35], higher religiosity was associated with a greater likelihood of discussing abstinence. However, higher religiosity was also associated generally with reduced discussions about contraception and condoms. Some previous research has found that family religiosity is linked to delayed timing of first sex, but also with lower contraceptive use when teens do become sexually active [46,47]. Additionally, teens who have ever had sex are much more likely to report talking to their parents about almost all SRH topics. This study relies on cross-sectional data, so we do not know the relative timing of parent-teen discussions and first sexual experience. This is an important limitation because in order for discussions to impact behavior, these discussions should happen before a teen first has sex and continue regularly once a teen has become sexually active in order to be effective at reducing risky sexual behaviors [48]. Research finds that speaking with children very early on about bodies, consent, and touching helps parents be supportive in helping their teen engage in healthy decision making [34].

Overall, we are cautiously optimistic about the recent increase in the prevalence of parent-teen conversations for males. While it remains to be seen whether these gains will persist into the future, we encourage researchers to explore what might be causing increases for boys but much less so for girls. We echo the recommendation of Robert and Sonenstein (2010) that clinicians, educators, and public health officials should encourage parent-teen conversations, so youth get the information they need. Federally funded teenage pregnancy prevention programs like the TPP and PREP programs, which have been funded since 2010, can be an important part of this effort. There has been an expansion of evidence-based teenage pregnancy prevention programs across the country in recent years [49]. These federal initiatives have focused efforts on youth in areas with high rates of teen pregnancy, and some of these programs focus on improving parent-teen discussions, either by intervening directly with parents [50,51] or by providing take-home assignments for teens to talk about with parents [52]. Federal initiatives have reached many teens in need of services. For example, TPP programs reached a half a million youth between 2010 and 2014 [25], and PREP reached 32,000 youth in 2015 [53]. While this analysis cannot assess the impact that these federal efforts may have had on any recent increases in parent-teen discussions about SRH, the increases in parent conversations, as well as the increased emphasis in the federal government funding mechanisms, may be indicative of a shift in the role that parents are playing in their teens’ lives. This possibility deserves further exploration as it is important these conversations be high quality and accurate. 

It is important to note, however, that not all education about sex for teens comes from parents. For some teens this is because their parents are not reliable sources of information, are not present in their children’s lives, or would not be willing to discuss these topics [54]. We need to ensure that these youth have other outlets and resources for this information. Health care providers, schools, community centers, and other adult mentors can also be sources of accurate, comprehensive, and non-judgmental information about sexuality and healthy relationships. 

Our study has several data limitations. One challenge is that, although we know the topic of SRH conversations, we do not know the content, quality, timing, or number of these conversations, which can be important for the effectiveness of these conversations [45,55]. Current initiatives to increase parent support for adolescent SRH focus on making sure that parents feel comfortable with the SRH content [52], on modeling communication [8], and on how to help parents not feel embarrassed or uncomfortable [34,56]. Additionally, we do not have data on some key factors that may influence parent-teen discussions, including economic background, social norms, age or gender of parent the child spoke to, parental religious beliefs, or immigrant status of the parent. The questions about talking to parents also ask about conversations before the age of 18, meaning that 15-17-year-old respondents may yet have these conversations. Finally, our analyses rely on teen reports of whether they had any discussions with their parents about SRH topics. We do not have any data on parents’ reports of these discussions, which may differ from teens’ reports [45,55]. For example, teens could interpret comments such as “be careful” as discussions about abstinence or STIs, while the parent intends them to be about contraception. More exploration of the content of parent-teen conversations would be valuable. 

## 5. Conclusions

This study has expanded on previous research by providing updated findings about the prevalence and differences in parent-teen SRH-related discussions across cohorts, and factors associated with SRH-related discussions. Our findings make us cautiously optimistic that parents are increasingly talking to their teens about important SRH topics. However, future research is needed to better understand the content of these discussions, the reasons behind the recent increases (particularly the large increases among Hispanics), whether these increases will be sustained, and whether these conversations are related to subsequent behavioral changes. Additionally, future research should examine ways to continue to support parent-teen SRH discussions in the future.

## Figures and Tables

**Table 1:** Weighted sample characteristics of female and male teens ages 15–19, across two cohorts of the National Survey of Family Growth, 2006–2015.

	Females in Cohort 1: 2006–2010	Females in Cohort 2: 2011–2015	Sig. Change across Cohorts	Males in Cohort 1: 2006–2010	Males in Cohort 2: 2011–2015	Sig. Change across Cohorts
**Percentage in Each Cohort**	52.9	47.1		52.7	47.3	
**Race/ethnicity**						
White	63.5	58.2		63.4	58.2	
Black	16.5	16.2		16.1	16.1	
Hispanic	20.0	25.6		20.5	25.8	
**High religiosity**	39.0	39.8		32.1	29.2	
**Disconnected (Neither working nor in school)**	10.1	7.4	*	9.1	7.2	
**Mother’s education**						
Less than high school	15.9	15.7		13.3	16.2	*
High school graduate or GED	31.1	28.1		34.1	27.6	
Some college or BA	53.1	56.2		52.6	56.2	
**Family structure at age 14**						
Lived with two parents (biological or adopted)	61.3	58.8		62.2	60.6	
**Age**						
15–17	55.1	58.0		60.5	60.9	
18–19	44.9	42.0		39.5	39.1	
**Ever had sex**	43.0	44.7		42.9	46.1	
**Total Sample Size (N)**	2076	1834		2148	1871	

Differences between cohorts with *p*-values < 0.05 are marked with an *.

**Table 2:** Prevalence of talking to parents about sexual and reproductive health (SRH) topics, by race/ethnicity, gender, and cohort.

Talk to Parents about…	Any SRH Topic			How to Say No to Sex			Contraception			STIs			How to Use a Condom		
***Female***	**2006–2010**	**2011–2015**		**2006–2010**	**2011–2015**		**2006–2010**	**2011–2015**		**2006–2010**	**2011–2015**		**2006–2010**	**2011–2015**	
**Total**	79.0	78.5		62.4	62.4		55.8	56.4		58.0	60.0		30.4	36.8	*
**Race/ethnicity**															
White	79.9	78.2		62.9	63.6		58.4	60.9		56.0	56.7		29.0	33.7	
Black	82.0	79.6		67.8	63.2		56.2	51.0		68.7	68.3		44.4	43.9	
Hispanic	73.8	78.6		56.6	59.2		47.2	49.6		55.5	62.2		23.3	39.5	*
				*a*			*a*	*a*		*a*	*a*		*a*	*a*	
**Nativity of Hispanics**															
U.S.-born	76.4	78.8		59.0	59.4		49.3	50.6		58.3	62.4		24.5	39.1	*
Foreign-born	63.7	77.5		47.3	58.1		38.8	44.6		44.1	61.0	*	18.7	41.1	*
	*b*									*b*					
***Male***	**2006–2010**	**2011–2015**		**2006–2010**	**2011–2015**		**2006–2010**	**2011–2015**		**2006–2010**	**2011–2015**		**2006–2010**	**2011–2015**	
**Total**	69.5	84.5	*	41.7	44.5		31.9	36.8	*	53.0	59.4	*	37.6	45.4	*
**Race/ethnicity**															
White	66.7	83.0	*	45.3	47.1		33.5	38.9		48.7	55.6	*	33.6	41.7	*
Black	78.2	86.5	*	43.3	46.5		28.0	34.6		65.7	66.4		50.5	58.7	
Hispanic	71.0	86.4	*	29.4	37.6	*	30.0	33.5		56.4	63.5	*	39.8	45.2	
	*a*			*a*	*a*					*a*	*a*		*a*	*a*	
**Nativity of Hispanics**															
U.S.-born	74.2	85.1	*	33.8	37.8		31.0	32.1		58.2	63.8		40.8	44.7	
Foreign-born	62.1	90.4	*	17.1	36.9	*	27.0	37.7		51.0	62.5		37.1	46.8	
	*b*			*b*											

Differences at the 5 percent level are marked with an *, an “a” or a “b”. * = significant differences across cohort; a = significant race/ethnicity differences; b = indicates significant differences across place of birth; The columns to the right of the percentages represent significant differences across cohorts while the rows below the columns indicate significant differences across race/ethnicity or nativity within cohorts.

**Table 3:** Odds ratios from logistic regression models predicting parent-teen discussions about sexual and reproductive health (SRH) among female teens (confidence intervals in parentheses).

Talk to Parents about…	Any SRH Topic	How to Say No to Sex	Contraception	STIs	How to Use a Condom
VARIABLES	Adjusted Odds Ratio	Adjusted Odds Ratio	Adjusted Odds Ratio	Adjusted Odds Ratio	Adjusted Odds Ratio
**2011–2015 Cohort** (ref: 2006–2010 cohort)	0.9	1.0	1.0	1.0	1.3 *
	(0.7–1.2)	(0.8–1.2)	(0.8–1.2)	(0.9–1.3)	(1.1–1.6)
**Race/ethnicity** (ref: white)					
Hispanic	1.0	0.9	0.7 ***	1.2	1.0
	(0.8–1.2)	(0.7–1.1)	(0.5–0.8)	(0.9–1.4)	(0.8–1.3)
Black	1.1	1.1	0.8 *	1.6 **	1.7 ***
	(0.8–1.6)	(0.8–1.4)	(0.6–1.0)	(1.2–2.1)	(1.3–2.2)
**High religiosity** (ref: religion is less important)	1.2	1.4 **	0.8 *	1.2	0.7 **
	(0.9–1.5)	(1.1–1.7)	(0.6–0.9)	(1.0–1.5)	(0.6–0.9)
**Disconnected** (ref: in-school or working)	0.5 ***	0.6 **	0.7 *	0.7 *	1.1
	(0.4–0.7)	(0.5–0.9)	(0.5–1.0)	(0.5–1.0)	(0.7–1.5)
**Lived with two parents at age 14** (ref: none or 1)	1.0	1.0	0.8	0.9	0.7 ***
	(0.8–1.3)	(0.8–1.2)	(0.7–1.0)	(0.7–1.1)	(0.6–0.9)
**Mother’s education** (ref: less than high school)					
High school graduate	1.3	1.0	1.2	1.1	1.1
	(0.9–1.7)	(0.7–1.3)	(0.9–1.7)	(0.8–1.4)	(0.8–1.5)
Some college or BA	1.3	1.3 *	1.2	1.1	1.0
	(1.0–1.7)	(1.0–1.6)	(0.9–1.5)	(0.9–1.5)	(0.8–1.4)
**Age**	0.8	0.9	1.0	0.8 *	0.9
	(0.7–1.1)	(0.7–1.1)	(0.8–1.1)	(0.6–1.0)	(0.8–1.2)
**Ever had sex**	1.8 ***	1.1	2.5 ***	1.8 ***	2.1 ***
	(1.4–2.2)	(0.9–1.3)	(2.1–3.1)	(1.5–2.2)	(1.7–2.6)
**Constant**	2.6 ***	1.4 *	1.1	1.0	0.4 ***
	(1.8–3.7)	(1.0–1.9)	(0.8–1.6)	(0.7–1.4)	(0.3–0.5)

Differences with *p*-values < 0.05 level are marked with an *, *p* < 0.01 level are marked with **, and *p* < 0.001 are marked with ***. The 95 percent confidence intervals are shown in parentheses.

**Table 4:** Odds ratios from logistic regression models predicting parent-teen discussions about sexual and reproductive health (SRH) among male teens (confidence intervals in parentheses).

Talk to Parents about…	Any SRH Topic	How to Say No to Sex	Contraception	STIs	How to Use a Condom
VARIABLES	Adjusted Odds Ratio	Adjusted odds ratio	Adjusted Odds Ratio	Adjusted Odds Ratio	Adjusted Odds Ratio
**2011–2015 Cohort** (ref: 2006–2010 cohort)	2.2 ***	1.2	1.2	1.2 *	1.3 **
	(1.7–2.9)	(1.0–1.4)	(1.0–1.5)	(1.0–1.5)	(1.1–1.6)
**Race/ethnicity** (ref: white)					
Hispanic	1.4 **	0.7 **	0.9	1.5 ***	1.2
	(1.1–1.8)	(0.5–0.9)	(0.8–1.2)	(1.2–1.8)	(0.9–1.5)
Black	1.5 *	0.9	0.7	1.6 ***	1.7 ***
	(1.1–2.0)	(0.7–1.1)	(0.6–1.0)	(1.3–2.0)	(1.3–2.2)
**High religiosity** (ref: religion is less important)	1.1	1.7 ***	0.9	1.1	0.8 *
	(0.9–1.4)	(1.4–2.1)	(0.7–1.1)	(0.9–1.3)	(0.6–1.0)
**Disconnected** (ref: in-school or working)	0.8	0.6 **	0.7 *	1.0	1.1
	(0.6–1.1)	(0.4–0.8)	(0.5–0.9)	(0.7–1.3)	(0.8–1.6)
**Lived with two parents at age 14** (ref: none or 1)	1.0	1.1	1.0	1.0	0.8
	(0.8–1.3)	(0.9–1.3)	(0.8–1.3)	(0.8–1.2)	(0.7–1.0)
**Mother’s education** (ref: less than high school)					
High school graduate	1.2	1.3	1.4 *	1.1	1.1
	(0.8–1.6)	(1.0–1.7)	(1.0–1.9)	(0.9–1.5)	(0.8–1.5)
Some college or BA	1.6 **	1.8 ***	1.8 ***	1.4 *	1.1
	(1.1–2.2)	(1.4–2.4)	(1.4–2.4)	(1.0–1.8)	(0.8–1.4)
**Age**	0.8	0.9	1.3 *	0.8 *	0.8 *
	(0.7–1.0)	(0.8–1.1)	(1.0–1.6)	(0.7–1.0)	(0.6–1.0)
**Ever had sex**	2.0 ***	1.0	2.1 ***	2.3 ***	3.2 ***
	(1.6–2.4)	(0.9–1.3)	(1.7–2.6)	(1.9–2.7)	(2.6–3.9)

Differences with a *p*-value < 0.05 level are marked with an *, *p* < 0.01 level are marked with **, and *p* < 0.001 are marked with ***. The 95 percent confidence intervals are shown in parentheses.

**Table 5:** Odds ratios from logistic regression models predicting parent-teen discussions among male and female Hispanic teens (confidence intervals in parentheses).

Talk to Parents about…	Any SRH Topic	How to Say No to Sex	Contraception	STIs	How to Use a Condom
VARIABLES	Adjusted Odds Ratio	Adjusted Odds Ratio	Adjusted Odds Ratio	Adjusted Odds Ratio	Adjusted Odds Ratio
**2011–2015 Cohort** (ref: 2006–2010 cohort)	1.7 ***	1.2	1.1	1.3 *	1.5 **
	(1.3–2.2)	(0.9–1.6)	(0.9–1.4)	(1.0–1.6)	(1.1–2.1)
**Foreign Born** (ref: Native born)	0.9	0.8	1.0	0.8	1.1
	(0.6–1.3)	(0.6–1.0)	(0.8–1.3)	(0.6–1.1)	(0.8–1.4)
**Female** (ref: male)	0.9	2.7 ***	2.2 ***	0.9	0.6 ***
	(0.7–1.1)	(2.2–3.3)	(1.7–2.7)	(0.8–1.2)	(0.5–0.8)
**High religiosity** (ref: religion is less important)	1.2	1.5 **	1.0	1.3 *	0.8
	(1.0–1.6)	(1.2–2.0)	(0.8–1.3)	(1.0–1.7)	(0.6–1.0)
**Disconnected** (ref: in school or working)	0.5 **	0.4 ***	0.6 **	0.5 ***	0.7 *
	(0.3–0.7)	(0.3–0.7)	(0.4–0.9)	(0.3–0.7)	(0.5–1.0)
**Lived with two parents at age 14** (ref: none or 1)	0.9	1.0	0.9	1.0	0.7 *
	(0.7–1.3)	(0.8–1.3)	(0.7–1.2)	(0.7–1.4)	(0.6–0.9)
**Mother’s education** (ref: less than high school)					
High school graduate	1.7 **	1.7 ***	1.6 **	1.2	1.7 ***
	(1.1–2.4)	(1.2–2.2)	(1.1–2.3)	(0.9–1.8)	(1.3–2.2)
Some college or BA	2.0 ***	1.9 ***	1.9 ***	1.4 *	1.2
	(1.4–2.7)	(1.4–2.4)	(1.4–2.6)	(1.1–1.9)	(0.9–1.6)
**Age**	0.7 *	0.9	1.0	0.8	1.0
	(0.6–1.0)	(0.7–1.2)	(0.8–1.4)	(0.6–1.1)	(0.7–1.2)
**Ever had Sex**	1.7 ***	0.8	1.9 ***	1.8 ***	1.8 ***
	(1.2–2.3)	(0.6–1.1)	(1.5–2.5)	(1.4–2.4)	(1.4–2.4)
**Constant**	2.1 ***	0.4 ***	0.2 ***	1.0	0.5 ***
	(1.4–3.1)	(0.3–0.5)	(0.2–0.3)	(0.7–1.3)	(0.3–0.7)

Differences with a *p*-value < 0.05 level are marked with an *, *p* < 0.01 level are marked with **, and *p* < 0.001 are marked with ***. The 95 percent confidence intervals are shown in parentheses.
